# Monitoring Health Effects of Vaping Discussed on Twitter in 2018 and 2019

**DOI:** 10.1007/s11606-021-06705-9

**Published:** 2021-04-09

**Authors:** Anuja Majmundar, Jon-Patrick Allem, Tess Boley Cruz, Jennifer B. Unger, Mary Ann Pentz

**Affiliations:** 1 Department of Preventive Medicine, Keck School of Medicine, University of Southern California, Los Angeles, CA USA; 2 American Cancer Society, DC Washington, DC, USA

## INTRODUCTION

The USA is currently witnessing a vaping epidemic. Despite the rising popularity of electronic nicotine delivery systems (ENDS) or e-cigarette use (vaping), there is a limited understanding of public perceptions about the health effects of these products. While scientific evidence about the long-term health effects is inconclusive, emerging findings suggest that vaping is linked to cancer, cardiovascular, respiratory, and other health issues.^1^ An understanding of public perceptions about the health effects of vaping can help identify priority areas for patient education in clinician settings. Social media surveillance of public perceptions of the health effects of vaping may inform patient-provider interactions, and complement findings from traditional survey research at a low cost.^2^ This study characterizes public conversations about the health effects of vaping on Twitter during 2018 and 2019.

## METHODS

Vaping-related posts (*n* = 2,516,664 posts in 2018, *n* = 2,731,399 posts in 2019, total *n* = 5,248,063) were obtained from January 1, 2018, to December 31, 2019, using Twitter’s Streaming Application Program Interface (API). Data cleaning procedures included removal of retweets so that each observation could be treated as an independent unit of observation, and removal of posts from bot accounts.^3^ Posts containing health-related references to e-cigarettes were identified using two dictionaries: (a) the Unified Medical Language System® (UMLS) Consumer Health Vocabulary (CHV), ^4^ comprising *n*=13,479 informal, common medical terms used by consumers and health care professionals; and (b) a list of *n*=177 additional informal terms corresponding to the CHV terms, wherever relevant (e.g., informal term of “inebriation” is “drunk”). Keywords from the two dictionaries encompassed health diseases and symptoms (referred to as “health effects” in this study). A total sample of *n* = 667,140 (*n* = 257,620 posts in 2018; *n* = 409,520 posts in 2019) e-cigarette-related posts with mentions of health effects were identified. Each post in the analytic sample was then classified to one or more of the 14 categories of health effects (see Table 1).

**Table 1:** Predominant Categories of Health Effects and Example Keywords

Health categories	Example keywords
Neurological	Coma, dizzy, lightheaded
Mental Health	PTSD, ADHD, jittery
Death	Die, kill, lost life
Injury	Injury, rupture, wound, bruise
Respiratory	Cough, wheeze, black lung
Pain	Painful, achy, cramping
Cancer	Cancer, tumor, malignant
Gastrointestinal	Belly, belch, vomit, puke
Cardiovascular	Stroke, heart attack, blood pressure
Weight-related	Fat, obese, weight, stoutness
Stress	Stressed, cortisol
Immunity	Flu, common cold, allergy
Pregnancy/In-utero	Pregnant, preggers, miscarriage
Other	Anemia, jaundice, mumps

## RESULTS

**Figure 1:**
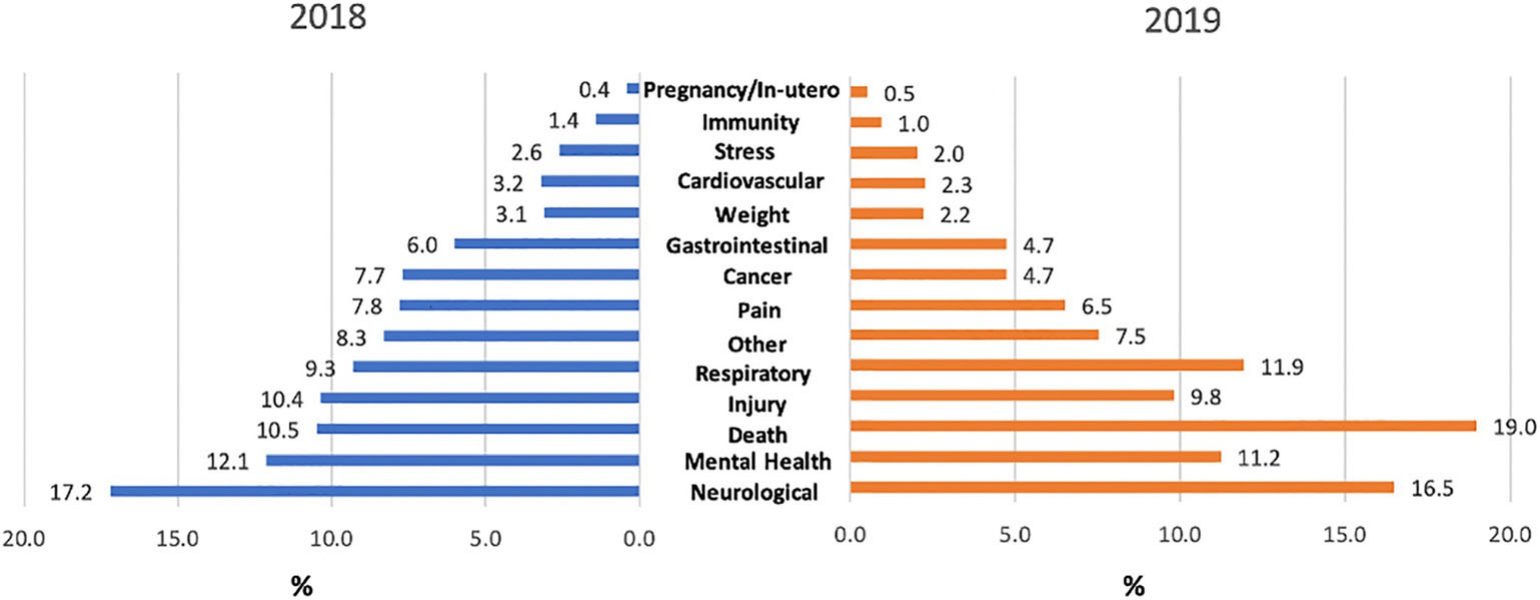
**Percentage distribution of categories of health effects during 2018 and 2019.**

Percentage distribution of categories of health effects by 2018 and 2019 is reported in Figure 1. In 2018, *Neurological* effects (*n* = 44,345; 17.2%) were the most common, followed by *Mental Health* (*n* = 31,227; 12.1%) and *Death *(*n* = 27,040; 10.5%). In 2019, *Death *was most commonly referenced (*n* = 77,719; 19.0%), followed by references to *Neurological* (*n* = 67,504; 16.5%) and *Respiratory *(*n* = 48,875; 11.9%) health effects in 2019. Compared to 2018, *Death*, *Mental health*, and *Respiratory* health effects were more prevalent in 2019. *Respiratory* health effects emerged as part of the top five categories in 2019.

## DISCUSSION

*Neurological, Death, Mental Health, and Respiratory *were all predominant categories of vaping-related health effects during 2018 and 2019 on Twitter. These findings may be relevant to primary healthcare providers as they counsel patients on the health risks associated with e-cigarette use.^5^ Armed with the insights from this study, clinicians can help patients better understand ways in which vaping may contribute to the abovementioned health effects. Chronic health conditions such as *Cancer*, *cardiovascular* diseases may also warrant consideration while discussing the potential long-term health risks of vaping since they may be less salient among patients. *Respiratory* health effects, a predominant category in 2019, may be potentially attributable to the outbreak of lung injuries associated with the use of vape products during that year.^6^ Findings will also inform future survey-based public health surveillance efforts and toxicology research examining long-term and short-term health effects of vaping.

This study drew data from public Twitter posts. Findings may not generalize to other social media platforms and may not represent data from individuals with private Twitter accounts. Specific references to the biological basis of the health effects (e.g., mentions of blocked arteries in the context of heart dysfunction) may not be captured in this study. While this study collected data from two different years, findings may not extend to other time periods.

Despite these limitations, findings may serve as a useful baseline to identify emerging categories and monitor predominant categories of perceived health effects of vaping in general and by source (e.g., news organizations, public health agencies) in the future. Unexpected peaks in reported categories of health effects may also help inform the surveillance of potential vaping-related outbreaks. Taken altogether, characterizing the public’s expressions of the health effects of vaping through Twitter data can offer insights to medical providers and inform survey-based research in the future.
